# Inadvertent Subdural Catheter Placement: A Rare Complication in Obstetric Anesthesia

**DOI:** 10.7759/cureus.27252

**Published:** 2022-07-25

**Authors:** Abdalhai Alshoubi, Danny Newhide

**Affiliations:** 1 Clinical Anesthesiology and Critical Care Medicine, St. Joseph’s Medical Center, Stockton, USA; 2 Anesthesiology and Critical Care, St. Joseph's Medical Center/Dignity Health, Stockton, USA

**Keywords:** subdural block, labor and delivery, epidural anesthesia, neuroaxial anesthesia, complications

## Abstract

Epidural neuraxial analgesia is a standard procedure for pain control during labor and delivery. One rare complication is accidental epidural catheter placement in the subdural space, a potential space between the arachnoid and dura membranes.

The incidence of the subdural blockade during neuraxial block is unknown. The subdural block suspicion arises when the clinical signs and symptoms do not fit epidural or subarachnoid local anesthetic injection. The clinical picture includes delayed or gradual onset, extensive sensory block with minimal motor block, hypotension more than an epidural neuraxial block, and less than spinal neuraxial block, and it can rarely track intracranially and causes dyspnea and loss of consciousness.

In this article, we report a case of inadvertent subdural catheter placement that was diagnosed clinically with unexpectedly high block involving the upper extremities. No radiological confirmation was used for the diagnosis.

## Introduction

Subdural placement of an epidural catheter is a rare complication that entails the placement of the epidural catheter in the subdural space - a potential space between the arachnoid mater and dura mater. It has a variable clinical presentation that makes it difficult to diagnose. The signs and symptoms depend on the spread of the anesthetic agent. The onset of the block is usually slow and gradual and can last for hours. There are no clear clinical guidelines for its management, and the treatment is supportive [1-6]. High vigilance of the clinical presentation leads to early detection and prevents further complications.

We report a case of inadvertent subdural catheter placement that was diagnosed clinically with unexpectedly high block involving both upper extremities. No radiological confirmation was used for the diagnosis. This report aims to increase the awareness of the signs and symptoms, risk factors, diagnosis, and management of subdural block during epidural catheter placement.

## Case presentation

The patient was a 23-year-old female gravida 1, para 0, American Society of Anesthesiologists (ASA) physical status class 2, presenting for elective cesarean section at 39 weeks for fetal macrosomia. She had no notable past medical history, and her pregnancy was complicated by obesity and mild gestational hypertension. The patient's height is 149 cm, and her weight is 87 kg with a body mass index of 39.2. Her airway examination revealed a good mouth opening, Mallampati grade 3, and a short neck with a reasonable range of movement. Pre-operative testing and exam were unremarkable.

The plan was made to proceed with combined spinal-epidural for surgical anesthesia. The first attempt at epidural placement was made in the intervertebral space between lumbar vertebrae 4 and 5 but failed to appropriately engage the ligamentum flavum at the midline with false loss of resistance at 7 cm as subsequent advancement abutted onto the bone, which was assumed to be lamina. A second attempt successfully engaged the ligament flavum appropriately and achieved a typical loss of resistance with saline at 8 cm. Upon coaxial introduction of a 27-gauge spinal needle, there was no spinal fluid return, so no spinal dose was administered. Another spinal neuraxial attempt was unsuccessful. The epidural catheter was threaded without difficulty and secured at the skin 3 cm past needle depth, which was 9 cm. Test dose with 3 ml of lidocaine 1.5% with 1:200,000 epinephrine was negative; 12 ml of lidocaine 2% with 1:200,000 epinephrine and 2 mL sodium bicarbonate 8.4% were then administered via incremental aspiration and injection through the epidural; her vital signs were within normal ranges as follows: blood pressure, 132/88 mm Hg; pulse, 88 beats/min; respiratory rate, 16 breaths/min; skin temperature, 97.6°F (36°C); and oxygen saturation, 99% on room air.

After 10 minutes, the patient reported only a mild decrease in cold sensation in the thoracic vertebra 6 level to sacral vertebra 3 level distributions, so an additional 5 ml of lidocaine 2% solution was given. It was planned to reassess the anesthetic level after draping for the surgical procedure, but no changes in her vital signs were noted. The reassessment done five minutes later revealed decreased sensation up to cervical vertebra 5 level with reduced grip strength and difficulty in lifting her legs bilaterally. Testing by the obstetric physician with forceps clamps on the skin before incision did elicit a sharp pain response from the patient. The decision was then made to convert to a general anesthetic. Rapid sequence induction was performed using 200 mg of intravenous (IV) propofol and 160 mg of IV succinylcholine followed by endotracheal intubation using a laryngoscope and endotracheal tube size 7.0 mm. At the end of the procedure, the patient was extubated and discharged to the labor floor where she had a complete resolution of her neuraxial anesthetic within 24 hours with no residual symptoms.

## Discussion

Subdural space is a potential space that is located between the dura membrane and arachnoid membrane. It extends from the brain meninges and ends at the lower border of the second sacral vertebra, and contains a small amount of serous fluid [7].

The distribution of the sensory block is usually bilateral, depends on the injected volume of local anesthetics, and usually spares the motor and sympathetic distribution explained by the subdural space anatomy [8]. For these reasons, the hemodynamic changes that happen with the subdural block are less apparent when compared to the subarachnoid. Its onset of action varies from seven to 30 minutes and can last up to three hours followed by full recovery [2].

The significant factors that increase the risk of accidental subdural block are difficult epidurals, combined spinal-epidural block, and aggressive handling of the needle, which lead to dura membrane tear and local anesthetic leak or catheter placement into the subdural space [9]. Altered anatomy from scaring due to back surgeries is another risk factor [2].

The diagnosis of the subdural block is done clinically. Lubenow et al. formulated two major and three minor clinical criteria to help diagnose subdural blocks. Major criteria included a negative cerebrospinal fluid aspiration test and unexpected high sensory block, while minor criteria included the delayed onset of action of local anesthetic for more than 10 minutes, a variable motor block, and extensive sympatholysis [2]. A subdural block is diagnosed in a patient with two major and at least one minor criterion. The subdural catheter misplacement can be confirmed radiologically with the use of contrast dye.

The management of the accidental subdural block is conservative, which includes removal of an epidural catheter and close monitoring of vital signs awaiting the resolution of symptoms.

## Conclusions

Inadvertent subdural catheter placement is a rare complication and usually presents with extensive and a typical sensory blockade compared to epidural neuraxial block. This is explained by the extension of the subdural space into the cranium. Fortunately, a majority of patients do not develop serious symptoms, and the treatment is supportive until the local anesthetic effect wears off. Clinicians' awareness of the potential signs and symptoms of the subdural block is essential for early diagnosis and managing this complication. Once a subdural block is identified, the catheter must be removed and placed at a different level.
